# 

**Published:** 1873-07

**Authors:** 


### Books and Pamphlets Received.

Report of Columbia Hospital for Women and Lying-in Asylum, Washington, D. C. By J. H. Tomphson, A. M., M. D., Surgeon-in-Chief. With an appendix. Washington: Government Printing Office, 1873.

Contributions to Medical Science, from the Bureau of Medicine and Surgery, United States Navy. A report on the origin and therapentic properties of Cundurango. By W. S. W. Ruschenberger, M. D., Medical Director U. S. N. Washington: Government Printing Office, 1873.

Mineral Springs of the United States and Canada, with analyses and notes of the prominent spas of Europe, and a list of sea-side resorts. By Geo. E. Walton, M. D. New York: D. Appleton & Co., 1873. Buffalo: Martin Taylor.

A Guide to Uniary Analysis, for the use of Physicians and students. By Henry G. Piffard, A. M., M. D. New York. Wm. Wood & Co., 1873. Buffalo; H. H. Otis.

Report of the Municipal Hospital, Philadelphia. (Comprising statistics of 2,373 cases of small pox.) By Wm. M. Welch, M. D., physician in charge.

Nævus. By J. H. Pooley, M. D., Yonkers, N. Y. Reprint from New York *Medical Journal*, June, 1873. New York: D. Appleton & Co.

Normal Ovanotoney. A paper read before the Georgia Medical Association. By Robert Battey, M. D.

Strictures of the Urethra. Results of operation with the dilating urethrotome with cases. By F. N. Otis, M. D. Reprint from New York *Medical Journal*, March, 1873. New York: D. Appleton & Co.

